# Sonic Hedgehog-Gli1 Signaling Pathway Regulates the Epithelial Mesenchymal Transition (EMT) by Mediating a New Target Gene, S100A4, in Pancreatic Cancer Cells

**DOI:** 10.1371/journal.pone.0096441

**Published:** 2014-07-29

**Authors:** Xuanfu Xu, Bin Su, Chuangao Xie, Shumei Wei, Yingqun Zhou, Hua Liu, Weiqi Dai, Ping Cheng, Fan Wang, Xiaorong Xu, Chuanyong Guo

**Affiliations:** 1 Department of Gastroenterology, the Tenth People's Hospital of Shanghai, Tongji University, Shanghai, China; 2 Department of Endocrinology, the Tenth People's Hospital of Shanghai, Tongji University, Shanghai, China; 3 Department of Gastroenterology, the Second Hospital of Zhejiang University, Hangzhou, Zhejiang Province, China; University of Nebraska Medical Center, United States of America

## Abstract

**Aims:**

The hedgehog signaling pathway plays an important role in EMT of pancreatic cancer cells, but the precise mechanisms remain elusive. Because S100A4 as a key EMT moleculer marker was found to be upregulated upon Gli1 in pancreatic cancer cells, we focused on the relationship between Shh-Gli1 signals and S100 genes family.

**Methods:**

On the base of cDNA microarray data, we investigated regulating mechanism of Gli1 to some members of S100A genes family in pancreatic cancer cell lines firstly. Then, the regulation of Gli1 to S100A4 gene was studied by molecular biology assays and the pro-metastasis effection of Gli1-dependent S100A4 was investigated in vitro. Finally, the expressions of Shh, Gli1, S100A4 and E-cadherin in pancreatic cancer tissues were studied by using immunohistochemistry assays.

**Results:**

Five members of the S100 genes family, S100A2, S100A4, S100A6, S100A11, and S100A14 were found to be downregulated significantly upon Gli1 knockdown. Gli1 enhancer prediction combining with in vitro data demonstrated that Gli1 primarily regulates S100A family members via cis-acting elements. Indeed, the data indicate S100A4 and vimentin genes were upregulated significantly by Shh/Gli1-expression increasing and E-cadherin was significantly reduced at the same time. Migration of PC cells was increased significantly in a dose-dependent manner of Gli1 expression (P<0.05) and siS100A4 significantly reversed the response of PC cells induced by L-Shh transduction (P<0.01).

**Conclusion:**

Our data establish a novel connection between Shh-Gli1 signaling and S100A4 regulation, which imply that S100A4 might be one of the key factors in EMT mediated by Shh-Gli1 signaling in pancreatic cancer.

## Introduction

Pancreatic cancer represents one of the leading causes of cancer-related mortality in industrialized countries [1]. The poor prognosis of this disease is mainly due to its early systemic metastasis [1]–[3]. A large number of studies have shown that the epithelial mesenchymal transition (EMT) might be a key mechanism of pancreatic cancer cells detaching from the primary tumor site and spreading to distant organs. However, the EMT-initiating signaling pathways remain elusive now in pancreatic cancer cells. Recently, hedgehog (Hh) signaling pathway has been demonstrated to be an important promoter of tumor growth in several gastrointestinal tract cancers [4], [5]. As to pancreatic cancer, it has been demonstrated that aberrant activation of Hh signaling pathway was resulted from sonic hedgehog (Shh) overexpression in the majority of cases [6]–[8]. A large number of studies have shown that the main mechanisms of Hh signaling pathway in cancer cells were to promote epithelial mesenchymal transition (EMT) process [9]–[11]. Moreover, it has been demonstrated that blocking this pathway significantly inhibited metastasis of tumor cells in vivo and in xenograft models [12], [13].

Since target genes of Gli1 were the key mechanisms of Hh signaling pathway, we attempted to understand its pro-metastatic mechanisms by identifing the targets of Gli1 in pancreatic cancer cells. As a key pro-metastatic target gene of Gli1 in pancreatic cancer it must have the following characteristics: First, there are effective Gli1 cis-acting elements in its gene sequence; Second, its transcription was regulated positively by Gli1; Third, it was upregulated in the majority of pancreatic cancer tissues and its expression level was positively correlated with Hh signaling; Fourth, its must be a key pro-metastatic functional factor. In previous study, we have performed a systematic research about the target gene profiles upon Gli1 in high-metastatic pancreatic cancer cell line through cDNA microarray and found that 5 members of this gene family were upregulated by Gli1. Especially, the S100A4 as a key moleculer marker promoting EMT process in pancreatic cancer was found to be upregulated more than 3 times in this study. On the base of these studies we focused on the relationship between Shh-Gli1 signals and S100 gene family.

In this study, we analyzed the Gli1 biding sites within DNA sequences of S100 genes family with bioinformatics tools and databases. Moreover, we attempted to identify new connections of Shh-Gli1 signaling pathway with S100 genes family and to demonstrate pro-metastatic function of S100A4 gene mediated by Shh-Gli1 signals in pancreatic cancer.

## Materials and Methods

### Cell cultures

The human PC cell lines, BxPC3, AsPC-1 and Panc-1, were all commercial cell lines and we obtained these cell lines from the Institute of Cytology Chinese Academy of Science.These cell lines were widely used in pancreatic cancer research [14]–[18]. Three cell lines were all cultured in RPMI-1640 supplemented with 10% fetal calf serum at 37°C in a humidified atmosphere of 5% CO2 in air.

### Vectors construction and cells infection

Lentiviral transfer vectors for human Gli1 shRNA or Shh cDNA were constructed by Genechem Co., Ltd, Shanghai, China. This system includes the lentiviral vector pLVTHM, the envelop plasmid pMD2G, and the packaging plasmid pRsv-REV and pMDlg-pRRE. The lentivirus-Shh (L-Shh) contained a 3.3-kb coding sequence for Shh and the lentivirus-Gli1i (L-Gli1i) contained Gli1 small hairpin RNA (5′-CTCCACAGGCATACAGGAT-3′) to the targeting sequence of the shRNA as previously described [19]. The lentivirus-control (L-C) which did not include the Gli1 interference sequences or Shh cDNA sequences served as control. Finally lentiviral constructs were verified via DNA sequencing to ensure accuracy. Recombinant lentivirus was produced by transient transfection of 293T cells following a standard protocol. When BxPC3, AsPC-1 and Panc-1 cells were about 50% confluent in RPMI-1640 containing 2% FCS, they were infected with the lentivirus constructs at MOI 5. The cells were harvested after 72 hours for further experiments. To identify functional L-Shh and L-Gli1i constructs, we routinely analyzed Shh and Gli1 expression by qRT-PCR and western-blotting.

### S100A4 Transient Knockdown

We used a RNAi sequence (siS100A4) and a negtive control sequence (siS100A4 MIS) which has shown efficient knockdown of S100A4 in human colon cancer HT29 cells in a previous report [20]. S100A4 knockdown was monitored by determining its mRNA and protein levels after 48 h upon siRNA transfection, respectively. And then the cells were used to transwell assays. Cells were lipofected with the siRNAs using Lipofectamine 2000 (Invitrogen) in according to the manufacturer's instructions.

### RNA extractions and real time RT-PCR assays

Total RNA samples were extracted with Trizol reagent (Invitrogen) according to the manufacturer's protocol and 100ng total RNA was reverse transcribed in 20 µl volume and 2 µl cDNA used for PCR according to the manufacturer's instructions. (TaKaRa Biotechnology, Dalian, China). The primer sequences were seen in table 1. CT (cycle threshold) values were standardized to CT values of GAPDH.

**Table 1:** The primer sequences for real time RT-PCR assays.

Gene	primer sequences
Gli1	F: 5'-TCTGCCCCCATTGCCCACTTG-3'
	R: 5'-TACATAGCCCCCAGCCCATACCTC-3'
Shh	F: 5'-CGGAGCGAGGAAGGGAAAG-3'
	R: 5'-TTGGGGATAAACTGCTTGTAGGC-3'
Patched1	5'-CGGCGTTCTCAATGGGCTGGTTTT-3'
	5'-GTGGGGCTGCTGTTTCGGGTTCG-3'
GAPDH	F: 5'-ACGGATTTGGTCGTATTGGG-3'
	R: 5'-TGGAAGATGGTGATGGGATT-3'
E-cadherin	F: 5'- CAATGCCGCCATCGCTTAC -3'
	R: 5'- CAAAATGCCATCGTTGTTCACT -3'
Vimentin	F: 5'- CAATGAGTCCCTGGAACGCC -3'
	R: 5'- CACGAAGGTGACGAGCCATT -3'
S100A2	F: 5'- CGGTCCAGGATGCCCAGTC -3'
	R: 5'- GGCTCCCAGGGTGAGGATTTAT -3'
S100A4	F: 5'- ACTCGGGCAAAGAGGGTGA -3'
	R: 5'- CTGGGCTGCTTATCTGGGAA -3'
S100A6	F: 5'- GGGGAGACTCGTCACCAGGC -3'
	R: 5'- GGTCCAAGTCTTCCATCAGCCT -3'
S100A10	F: 5'- GCCGCCTCCCTCTACCCAC -3'
	R: 5'- GCCTTTATCCCCAGCGAATTT -3'
S100A11	F: 5'- AGGGCGTGGGTTGAGGAGA -3'
	R: 5'- TGTGGAGATGATGACAGAAAGGC -3'
S100A13	F: 5'- GCTGGGAGGAGCGGTTAGA -3'
	R: 5'- GCAAAGGTGAAGAAGGTGGTGA -3'
S100A14	F: 5'- CAGTGTCGGTCAGCCAACG -3'
	R: 5'- TCCCAGAAACTCCTGAACTCCA -3'
S100A16	F: 5'- TGAACTGGGGTCCCTTTGTG -3'
	R: 5'- TCAGGACGGACCCAGAATCA -3'
S100P	F: 5'- AAGAGGCTGCCAGTGGGACA -3'
	R: 5'- TTGGTCAGGGTCTGCGTGC -3'
TCHH	F: 5'- TTCGGAGACCACATGACCCTA -3'
	R: 5'- GCTCTTCCCGTTCTTGCCA-3'

### Protein extractions and western-blotting assays

Total protein samples were extracted with RIPA buffer according to the standard method and the samples were normalized for protein content by a commercially available kit (Bio-Rad Company). Equal amounts of protein were used for western-blotting assays according to the standard protocol for Shh, Gli1, S100A4, E-cadherin, VIM (vimentin) and GAPDH detection.

### Transwell assays

Cell invasion assay-24 samples kits (Chemicon, Bedford, MA) were used to study the invasion/migration of PC cell lines. After 24h incubation, migration counts of PC cells were obtained by photographing the membrane through the microscope. Counts were taken in 5 highest cells areas at a high power magnification (×200). The mean value of the fields counted was considered as the migration count of PC cells.

### The Formaldehyde Cross-linking Chromatin Immunoprecipitation (XChIP) assays

The XChIP assays were carried out as the previous studies [21]. In brief, the chromatin of AsPC-1 cells (3×10^7^) was collected with 1 mL IP buffer containing protease inhibitor cocktails. Chromatin was sheared by using a sonicator in an ice box (6 rounds of 10s pulses, 350 W, 60 second intervals) and Crosslinking was reversed by adding 20 µL of 5 M NaCl overnight at 65°C. DNA was extracted using phenol/chloroform assay. 20 µL of DNA was electrophoresed on a 1.5% agarose gel and the rest was used as INPUT DNA. ChIP-grade goat polyclonal Gli1 antibody (0.1 µg/mL) (Santa Cruz Company) was used for immunoprecipitation, the mouse IgG (0.1 µg/mL) (Santa Cruz Company) was added as a random control, RNA polymerase II antibody (0.1 µg/mL) as a positive control and β-actin antibody (0.1 µg/mL) as a negative control. The primer of the promoter region of GAPDH (Santa Cruz Company) was used as a negative control. (Table 2).

**Table 2:** The primers for XChIP assays.

Gene	primer sequences
S100A4	F: 5'-GCTGTGGCACTTACCGCATC-3'
	R: 5'-CCTCCGTCTTACGTGCATGTG-3'
S100A6	F: 5'-GCCCTCCTTGCCCACATTG-3'
	R: 5'-TCTTCGTGACACGTGACTCGG-3'
S100A2	F: 5'-GTGCTGAAGTGTTGACTGAAGGG-3'
	R: 5'-GGCAGGAGAATGGCGTGAA-3'
GAPDH	F: 5'-TACTAGCGGTTTTACGGGCG-3'
	F: 5'-TCGAACAGGAGGAGCAGAGAGCGA-3'

### Luciferase reporter vectors assays

The S100A4 promoter luciferase reporter vectors were constructed by Genechem Co., Ltd, Shanghai, China. Briefly, a 1.5-kb cDNA fragment (from −766 to +734 of human S100A4 gene) with XhoI and HindIII sites was synthesized and cloned into the Xho I and Hind III sites of the pGL3 basic luciferase vector to produce pGL3-1.5 S100A4 containing three Gli1 biding sites [ACCCACCAC (−349∼−357), TGGGTGGTG (−227∼−235) and CTGGTGGGG (126∼134)]. In the mutagenesis vectors, Gli1 potential binding sites sequences were replaced respectively by GATTCTTAA sequence that has no known binding sites for any transcription factors [22]. The single Gli1 biding site mutagenesis vectors included pGL3-1.5 S100A4 Mut 1 [GATTCTTAA (−349∼−357), TGGGTGGTG (−227∼−235) and CTGGTGGGG (126∼134)], pGL3-1.5 S100A4 Mut 2 [ACCCACCAC (−349∼−357), GATTCTTAA (−227∼−235) and CTGGTGGGG (126∼134)] and pGL3-1.5 S100A4 Mut 3 [ACCCACCAC (−349∼−357), TGGGTGGTG (−227∼−235) and GATTCTTAA (126∼134)]. The multiple Gli1 binding sites mutagenesis vectors included pGL3 1.5 S100A4 Mut 1–2 [GATTCTTAA (−349∼−357), GATTCTTAA (−227∼−235) and CTGGTGGGG (126∼134)], pGL3 1.5 S100A4 Mut 1–3 [GATTCTTAA (−349∼−357), TGGGTGGTG (−227∼−235) and GATTCTTAA (126∼134)] and pGL3 1.5 S100A4 Mut 2–3 [ACCCACCAC (−349∼−357), GATTCTTAA (−227∼−235) and GATTCTTAA (126∼134)]. The final vector constructs were verified via DNA sequencing to ensure accuracy. AsPC-1 cells were transfected with 5 µg of either the pGL3-1500 S100A4 or the pGL3-1500 S100A4 mutant constructs and 2 µg of the renilla luciferase construct (pGL4.75, Promega) as an internal control for normalization using the Lipofectin reagent method protocol. 48 hours after transfection, AsPC-1 cells were used for the luciferase assays according to the protocol from the dual luciferase reporter kit (Promega) and the volues were readed using a luminometer. Each experiment was repeated at least 3 times, each time in triplicate.

### Patient samples

In this work, 102 banked paraffin-embedded cancer tissues from different PC patients (from 63 men and 39 women, mean age 56.7 years, range 44–75 years) were analyzed by immunohistochemistry. All patients have agreed this research. All samples were obtained from the tenth people's hospital of Tongji university, China and all patients have signed a consent document, in which they agreed that the surgical excision of malignancy would be used in medical research before their operations. The ethics committees of the Tongji Universities approved the consent procedure and the studies.

### Immunohistochemistry assays

The paraffin-embedded PC tissues were used for identification of Shh, Gli1, S100A4 and E-cadherin. Shh polyclonal antibody was purchased from R&D Company and the others (Gli1, S100A4 and E-cadherin) were from Sant Cruz Company. The immunohistochemistry assays were carried out as the preceding studies and the expression levels of Shh, Gli1, S100A4 and E-cadherin genes were graded based on previously described guidelines [23].

### Statistical Analysis

For all statistical analyses, a software package SPSS17.0 (SPSS, Inc, Chicago, IL, USA) was used. Continuous variables were expressed as means ± SE and a non-paired Student's t-test was used for statistical evaluation. The correlations between expression of Shh, Gli1, S100A4 and E-cadherin proteins and the relationship between the clinical characteristics of the patients and the five proteins were analyzed using the Spearman rank test. *P*<0.05 was considered to be statistically significant.

## Results

### Differential expression of S100 gene family upon Gli1 in AsPC-1 cells

**Figure 1:**
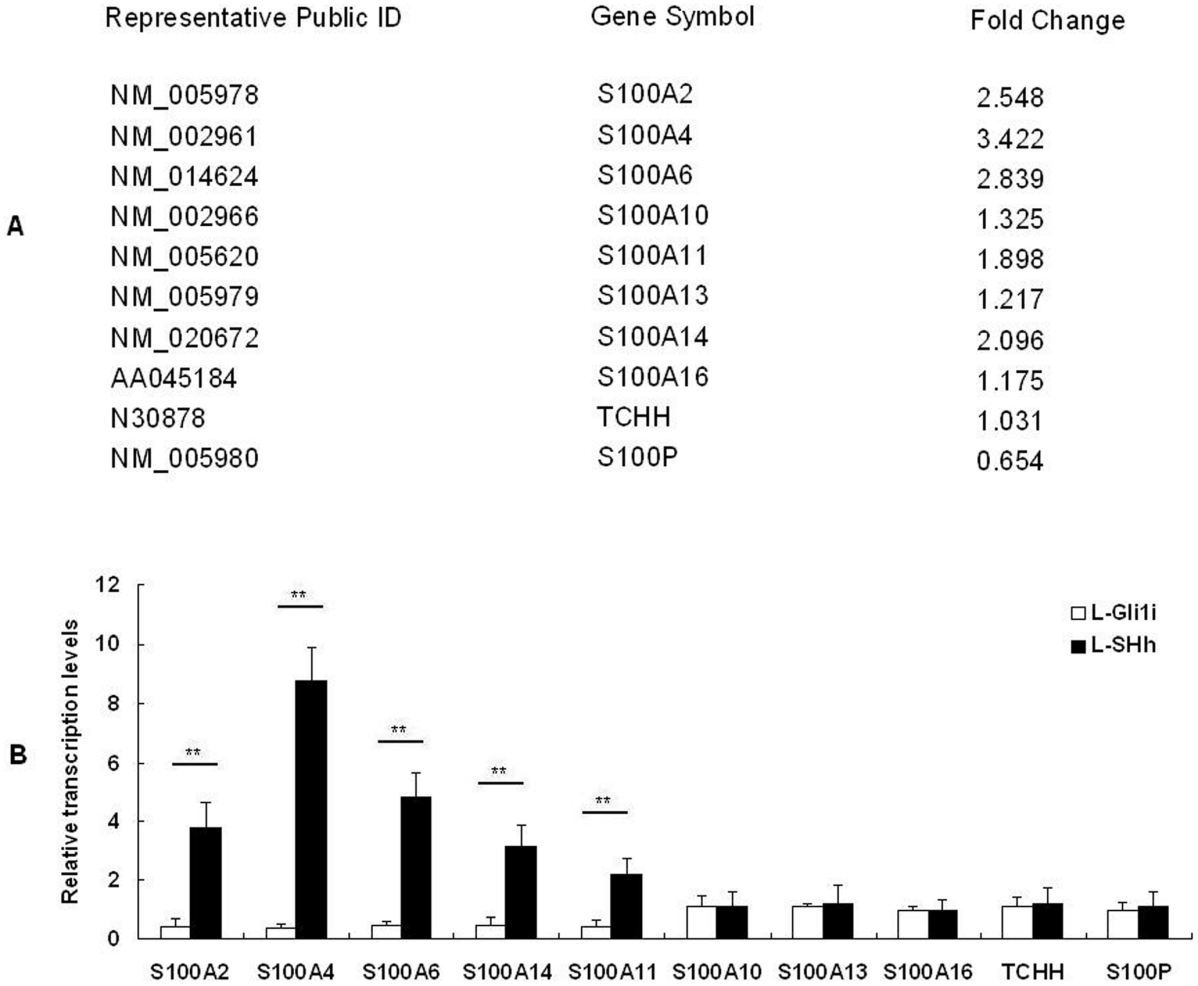
Expression of the S100 gene family. A: cDNA microarray data about S100 gene family; B: The differential expression levels of partial members of S100 gene family by qRT-PCR. The expression of GAPDH is as a control. * *P*<0.05, ** *P*<0.01.

To delineate the gene expression induced by Shh-Gli1 signals in pancreatic cancer cells, AsPC-1 cells were used to cDNA microarray assays comparing lentivirus-control vs lentivirus-Gli1i cells. When focusing on the EMT-related genes in cDNA data, we found the positive relationship between Shh signals and S100 genes family[21]. In our screen (the expression signals included 23 members of S100 genes family except for S100A17 and S100A18) 10 genes, S100A10, S100A11, S100A13, S100A14, S100A16, S100A2, S100A4, S100A6, S100P and TCHH, were expressed and 13 genes, S100A1, S100A12, S100A3, S100A5, S100A7, S100A15, S100A8, S100A9, S100B, S100G, S100Z, RPTN and FLG were not expressed in AsPC-1 cells. Comparison analysis from lentivirus-control group vs lentivirus-Gli1i group cells showed that S100A2, S100A4, S100A6, S100A11 and S100A14 were upregulated upon Gli1. (Figure 1A). The ten genes, S100A10, S100A11, S100A13, S100A14, S100A16, S100A2, S100A4, S100A6, S100P and TCHH, were picked for validation by real-time RT-PCR. As showed in Figure 1B, S100A2, S100A4, S100A6, S100A11 and S100A14 were found to be upregulated, with no significant changes in the other five genes. These data are consistent with that obtained from the cDNA microarray.

### Predicted Gli1 biding sites and putative Gli1 target genes within S100A gene family

To further study the mechanism of Shh-Gli1 signaling pathway on the regulation of the S100 gene family, we downloaded the DNA sequences of S100A genes from Gene Bank and analyzed homologous sequences of Gli1 biding site (GACCACCCA) by utilizing Blastz bioinformatics tool. The bioinformatics data showed that almost all members of S100A gene family contain many highly conserved homologous sequences (the difference is no more than one base) and these putative Gli1 biding sites exhibited the characteristics of the cluster arrangement. The Table 3 showed the putative Gli1 biding sites within the proximal regulatory region containing intergenic regions, intragenic regions, untranscripted regions and untranslated regions (no more than 5.0 kb away from TSS). The data probably confered a somewhat higher overall degree of evidence that these candidates are in fact differentially expressed in the setting of pancreatic cancer, possibly as downstream targets of an aberrantly reactivated.

**Table 3:** The putative Gli1 biding sites in S100A gene family.

Gene	Site	Sequence	Homology
S100A1	350∼358	ATGGTGGGT	89%
	1415∼1423	CTGGTGGGT*	100%
	1780∼1788	GGGGTGGTC	89%
S100A2	−4932∼−4940	TGGGTGGTA	89%
	−3703∼−3711	ACCCCCCAG	89%
	−3323∼−3331	TGGGTGGGC	89%
	−2870∼−2878	AGCCACCAG	89%
	−2366∼−2374	CTGGGGGGT	89%
	64∼72	TGGGTGGGC	89%
	326∼334	CTGGTGAGT	89%
	418∼426	CTGGTGGGA	89%
S100A3	−4900∼−4908	ACCCTCCAG	89%
	−3205∼−3213	GACCACCCA*	100%
	−1567∼−1575	ACCCACAAG	89%
	491∼499	TGGGTGGTG	89%
S100A4	−349∼−357	ACCCACCAC	89%
	−227∼−235	TGGGTGGTG	89%
	126∼134	CTGGTGGGG	89%
S100A5	−947∼−955	GTGGTGGGT	89%
	424∼432	CTGGTGGGT*	100%
S100A6	−246∼−254	CTGGTGGGG	89%
	786∼794	TGGGTGGTT	89%
S100A7	−4794∼−4802	ATGGTGGGT	89%
	−4276∼−4284	TGGGTGTTC	89%
	−4141∼−4149	CACCACCCA	89%
	−3686∼−3694	CACCACCCA	89%
	−2717∼−2725	CTGGTGGCT	89%
	−99∼−107	ACCCACCTG	89%
S100A8	−4865∼−4873	ACCCACCAC	89%
	−3944∼−3952	ACCCACCTG	89%
	−218∼−226	GACCACCAA	89%
	484∼492	TCCCACCAG	89%
S100A9	−4428∼−4436	GACCACCCC	89%
	−3295∼−3303	TGGGTGGGC	89%
	−3169∼−3177	ACCCACCTG	89%
	−2294∼−2302	CTGGTGGGG	89%
	−793∼−801	CTGGTGGGC	89%
S100A10	−3501∼−3509	ACCCACTAG	89%
	383∼391	ACCCACCCG	89%
	522∼530	TGGGTAGTC	89%
	617∼625	CTGGTGGGG	89%
	784∼792	TTGGTGGGT	89%
	2638∼2646	TACCACCCA	89%
	3051∼3059	TGGGTGGGC	89%
S100A11	−2685∼−2693	CGAACACCCA	89%
S100A12	−2510∼−2518	TGGGTGCTC	89%
	−2048∼−2056	CTTGTGGGT	89%
	−225∼−233	CGGGTGGGT	89%
	78∼86	TAGGTGGTC	89%
S100A13	−1340∼−1348	AGGGTGGTC	89%
	−1305∼−1313	ACCCACCTG	89%
	3400∼3408	GGGGTGGTC	89%
	3731∼3739	CTGGTGGGT	100%
	4660∼4668	ATGGTGGGT	89%
S100A14	−1656∼−1664	TGGGTGCTC	89%
	138∼146	TGGGTGGTT	89%
S100A15	−1620∼−1628	GTGGTGGGT	89%
	1233∼1241	ACCCCCCAG	89%
S100A16	−483∼−491	CTGATGGGT	89%
	110∼118	TGGGAGGTC	89%
	270∼278	CTGGGGGGT	89%
	448∼456	GGGGTGGTC	89%
	2194∼2202	CTGGTGGGG	89%
	2682∼2690	CACCACCCA	89%

### The new target gene and its effective Gli1 binding sites identification in AsPC-1

**Figure 2:**
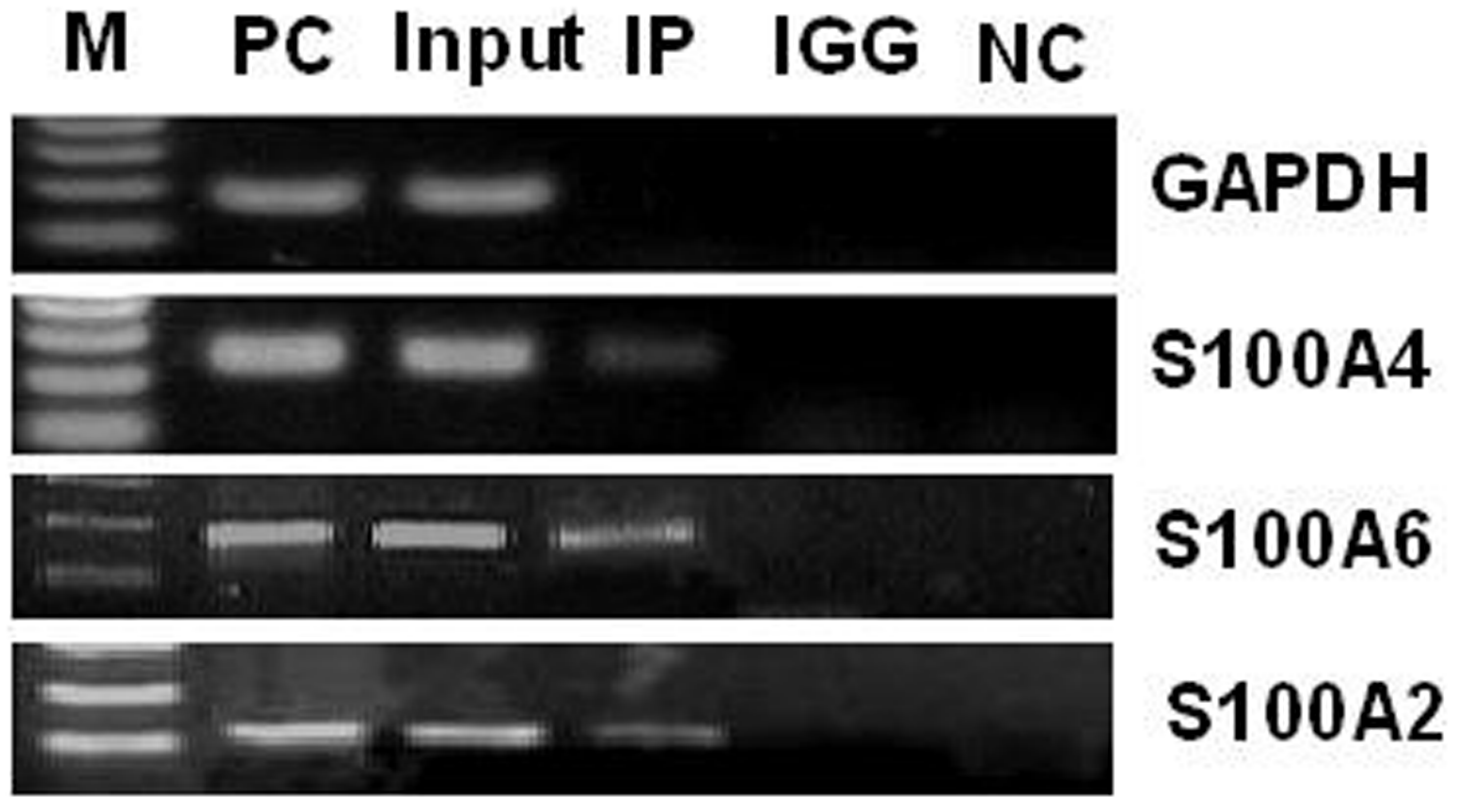
Binding of Gli1 to promoter region of S100A2, 4 and 6 genes in AsPC-1 cells analyzed by XChIP-PCR assay. M: DNA Marker; PC: RNA polymerase II antibody for XChIP positive control; IP: Gli1 XChIP; IGG: mouse IgG for XChIP random control; NC: β-actin antibody for XChIP negative control.

The PCR primers for XChIP assays were designed according to 1.5kb-long promoter sequences of S100A2, A4 and A6 genes, which containing several predicted Gli1 biding sites (64∼72, 326∼334, 418∼426 for S100A2; −349∼−357, −227∼−235, 126∼134 for S100A4; −246∼−254, 786∼794 for S100A6. the homology of each site is 89%.). (Table 3). The results of the XChIP assays showed that transcription factor Gli1 bound to the promoters of S100A2, 4 and 6 genes respectively in the cultured AsPC-1 cells (Figure 2).

**Figure 3:**
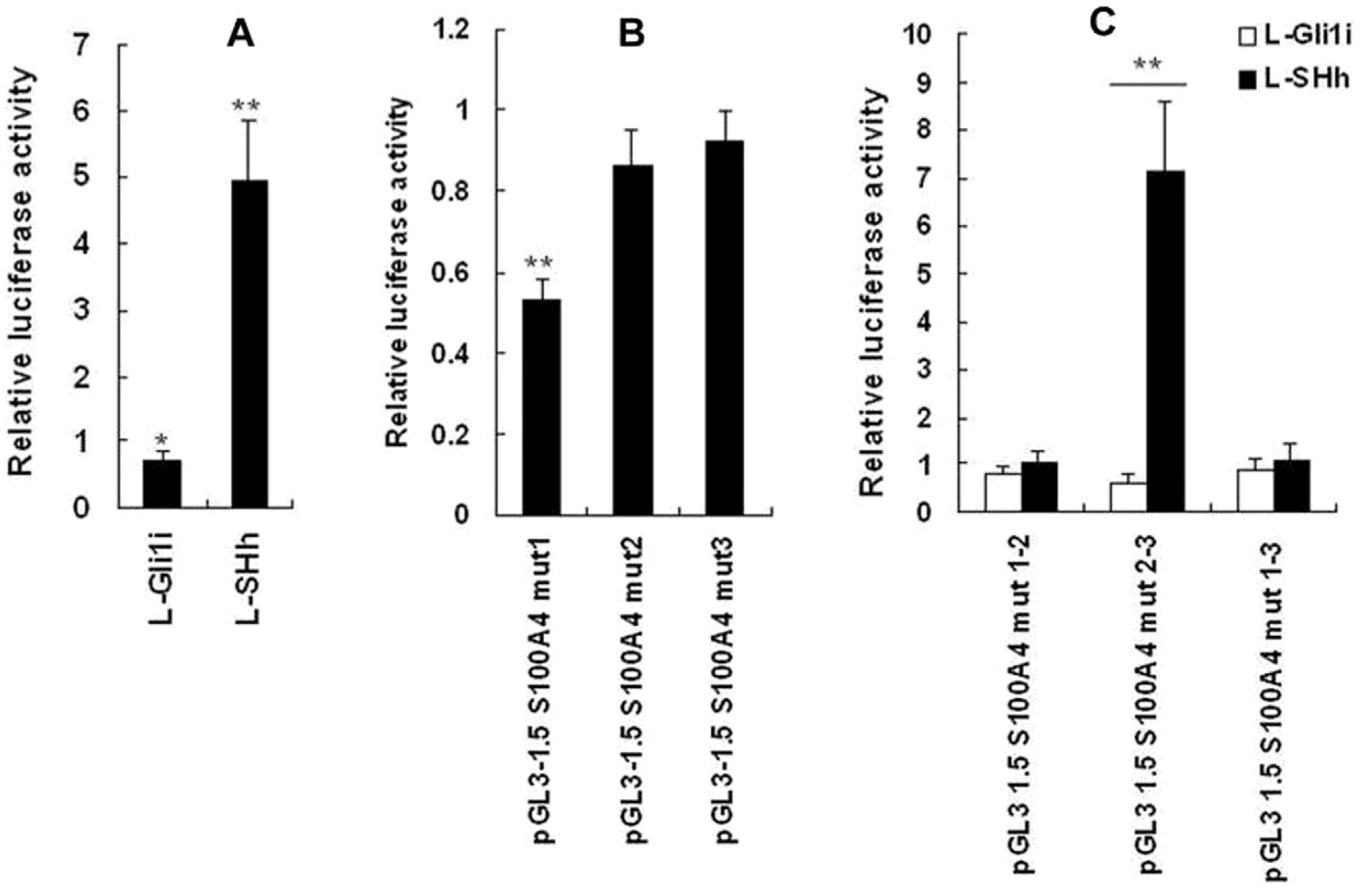
Identification of effective Gli1 binding sites on the S100A4 promoter. A: S100A4 luciferase activity increases with increasing expression level of SHh in AsPC-1 cells. The pGL3-1.5 S100A4 and Renilla luciferase vectors were transiently transfected into L-Gli1i infected, L-C infected or L-Shh infected AsPC-1 cells respectively. Results were normalized for transfection efficiency using Renilla luciferase and the L-C infected group was arbitrarily given a value of 1. B: Relative luciferase activity of the S100A4 promoter Gli1 binding site mutants. AsPC-1 cells transfected by L-Shh were transiently transfected 5mg of each reporter construct including pGL3-1.5 S100A4, pGL3-1.5 S100A4 Mut1, Mut2 and Mut3 respectively. The pGL3-1.5 S100A4 was arbitrarily given a value of 1 and the activities of the other transfections were adjusted relative to this activity. C: Site 1 was responsible for Gli1 transcription. Relative luciferase activity from different AsPC-1 cell groups, L-Gli1i infection, L-Shh infection or L-C infection, transfected by different constructs, pGL3 1.5 S100A4 Mut 1–2, Mut 2–3 and Mut 1–3 with Renilla luciferase vectors. Results were normalized for transfection efficiency using Renilla luciferase and the L-C infected group was arbitrarily given a value of 1. * *P*<0.05, ** *P*<0.01.

Results of luciferase assays showed that promoter activities of pGL3-1500 S100A4 increased with expression levels of Gli1 gene in a dose dependent manner (*P*<0.05). The pGL3-1.5 S100A4 Mut 2 or 3 had no significant effect on luciferase activity (*P*>0.05), however, pGL3-1.5 S100A4 Mut 1 decreased luciferase activity significantly compared with pGL3-1.5 S100A4 (*P*<0.05), which suggest that site 1 might be enhancers of Gli1. The luciferase activity of plasmids contains site 1 increased significantly (*P*<0.01) and that of plasmids contain site 2 or 3 did not change significantly (*P*>0.05). (Figure 3A, B, C). The sequence of site 1, ACCCACCAC, had also been demonstrated to be recognized and sis-activated by Gli1 proteins previously [24], [25].

### Regulation of Shh-Gli1 signals to expression of S100A4 and to invasion/migration of PC cells through mediating S100A4 in vitro

**Figure 4:**
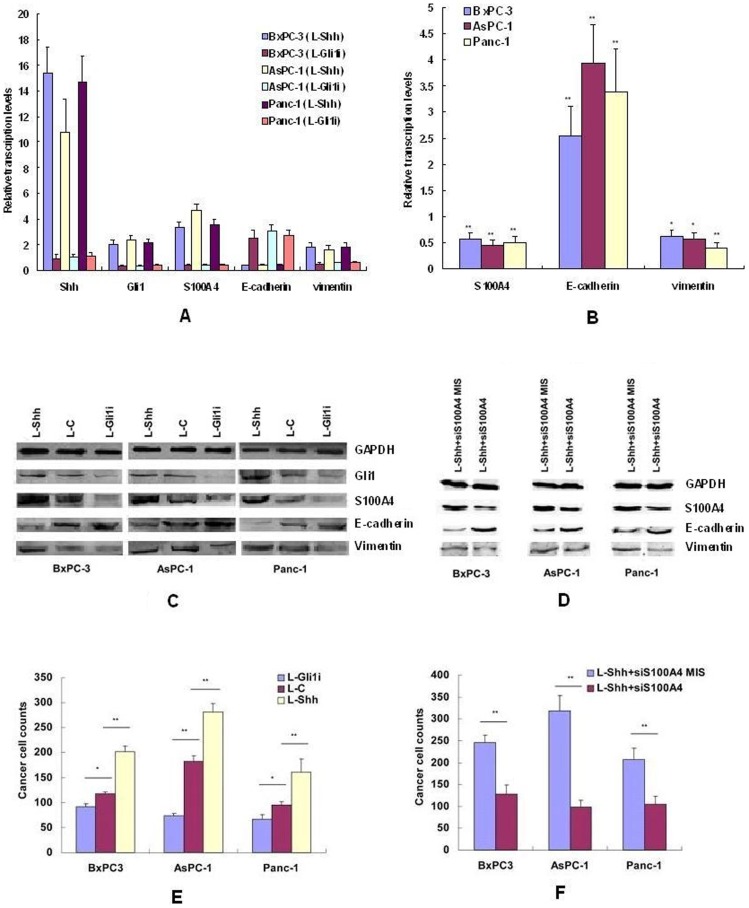
Hh signaling pathway promoting invasion/migration of pancreatic cancer cells through mediating S100A4. A: The relative transcription levels of Shh, Gli1, S100A4, E-cadherin and vimentin were regulated by L-Gli1i/L-Shh transduction. B: The relative transcription levels of S100A4, E-cadherin and vimentin in L-Shh transfected cells were reversed by siS100A4 transduction. C: The expression levels of Gli1, S100A4, E-cadherin and vimentin proteins regulated by L-Gli1i/L-Shh transduction. D: The expression levels of S100A4, E-cadherin and vimentin proteins were reversed by siS100A4 transduction. E: The invasion/migration of pancreatic cancer cells regulated by L-Gli1i/L-Shh transduction were analyzed by transwell assays. F: The invasion/migration of L-Shh transfected cells were reversed by siS100A4 transduction. * *P*<0.05, ** *P*<0.01.

To further study the pro-metastasis function of Gli1-derived S100A4 in pancreatic cancer cells, five groups of PC cells, L-Gli1i, L-C, L-Shh, L-Shh+siS100A4 MIS and L-Shh + siS100A4, were used to analize the regulation of Shh-Gli1 signals to S100A4, E-cadherin and VIM genes by real time RT-PCR and western-blotting assays. And then the same five group cells were used to evaluate invasion/migration of PC cells *in vitro* by transwell assays. The results of qRT-PCR and western-blotting showed that expression levels of S100A4 and VIM genes were increased significantly by Shh/Gli1-expression increasing. In contrast, the expression levels of E-cadherin were significantly reduced at the same time. (Figure 4A, C). Moreover, the S100A4 knocked-down signifiantly reversed downregulated E-cadherin and upregulated VIM induced by L-Shh transduction. (Figure 4B, D). The results of transwell assays showed the migration of PC cells were significantly decreased in L-Gli1i group and increased in L-Shh group respectively compared with L-C group (*P*<0.05). (Figure 4E). Moreover, siS100A4 significantly reversed the response of PC cells induced by L-Shh transduction (*P*<0.01). (Figure 4F). Thus, it seems that S100A4 mediated by abnomal activated Shh-Gli1 signaling pathway could be one of key pro-migration factors in pancreatic cancer cells.

### Correlation between the expression of Shh, Gli1, S100A4 and E-cadherin proteins in PC tissues

**Figure 5:**
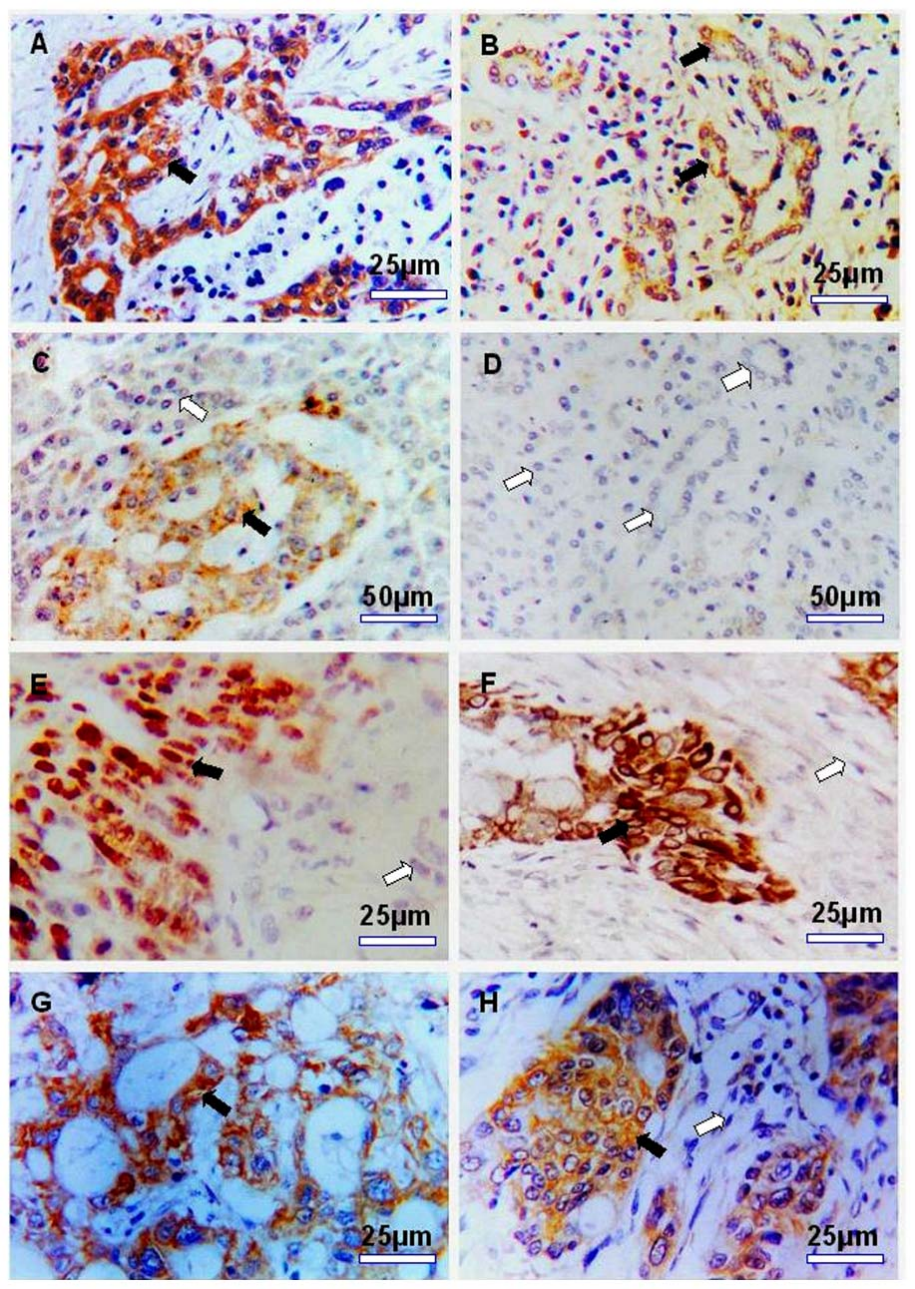
Expression of Shh, Gli1, S100A4 and E-cadherin in pancreatic cancer tissues analyzed by immunohistochemistry. A: Strong cytoplasmic staining for Shh in pancreatic cancer cells (×400); B: Weakly positive staining for Shh in ductal complex of chronic pancreatitis tissues (×400); C: Weakly positive staining for Shh in islet cells of normal cancer side tissue (×200); D: Negtive staining for Shh in normal ductal epithelial/acinar cells of cancer side tissue (×200); E: Cancer cells show strong nuclear staining for Gli1 and normal cancer side tissues were negtive (×400); F: Strong positive perinuclear cytoplasmic and part nuclear staining for Gli1 in pancreatic cancer cells (×400); G: Strong positive cytoplasmic staining for S100A4 in pancreatic cancer cells (×400); H: Positive cytoplasmic staining for E-cadherin in pancreatic cancer cells. Black arrows point to positive staining area and white arrows point to negtive staining area.

The results of immunohistochemistry showed that the positive stainings of Shh. Gli1 and S100A4 proteins localized almost exclusively to the neoplastic cells beside weakly positive staining of Shh in the islet cells and the chronic pancreatitic duct complex occasionally being seen in pancreatic tissues apart from cancer. In contrast, the protein expression of E-cadherin was markedly downregulated in PC tissues compared with normal tumor-adjacent tissues. The staining patterns of Shh and S100A4 were diffuse cytoplasmic type, that of E-cadherin was diffuse cytomembrane type and that of Gli1 display perinuclear cytoplasmic and nuclear staining. Because the Gli1 was activated only after entering the nucleus, the samples with only cytoplasmic staining were counted as negative expression. (Figure 5). Immunoreactivity ratios were 78.43% for Shh (80 of 102), 51.96% for Gli1 (53 of 102), 81.37% for S100A4 (83 of 102) and 48.04% for E-cadherin (49 of 102) in PC samples. The Spearman rank test analysis showed that significant positive correlations were noted between Shh, Gli1 and S100A4 at the protein level in PC (Shh vs Gli1: CC = 0.697, *P*<0.01; Shh vs S100A4: CC = 0.476, *P*<0.01; Gli1 vs S100A4: CC = 0.510, *P*<0.01). Conversely, the significant negative correlations between the three proteins and E-cadherin were confirmed (Shh vs E-cadherin: CC = −0.278, *P*<0.01; Gli1 vs E-cadherin: CC = −0.207, *P*<0.05; S100A4 vs E-cadherin: CC = −0.285, *P*<0.01).

### The correlation between the expression of four proteins and clinicopathological features of the PC patients

The statistical analysis data showed that expression levels of Shh (CC = 0.245, *P*<0.05 and CC = 0.254, *P*<0.05) and S100A4 (CC = 0.265, *P*<0.01 and CC = 0.220, *P*<0.05) proteins were positively correlated with tumor size and lymph node metastases and that of Gli1 protein was positively correlated with lymph node metastases (CC = −0.370, P<0.01), while that of E-cadherin protein was negtive correlated with lymph node metastases (CC = −0.203, *P*<0.05). However, no significant correlation was found between the four proteins and age, gender, tumor location and differentiation.

## Discussion

During tissue morphogenesis activation of Hh signaling pathway almost accompany the occurrence of EMT and this process is required for migration of Hh-responsive cells [26], [27]. It was reported that the mechanisms of Shh-Gli1 signaling pathway promoting malignant tumors involved in many aspects of malignant biological behaviors but its main role in tumors is to promote EMT and maintain tumor stem cells [28]. In this study, we provide a systematic research about the mechanism of Gli1 in a high-metastasis pancreatic cancer cell line, AsPC-1. The AsPC-1 cell line contains both Ki-ras gene mutation and DPC4 gene deletion, which were typical pancreatic cancer molecular pathological change [29], [30]. Moreover, the expression level of Gli1 was knocked down moderately by using the RNA interference system, which was more consistent with the actual molecular pathological change of pancreatic cancer than artificial gene knockout. So the target gene spectrum obtained in our screening was more consistent with the actual situation of pancreatic cancer cells.

To date, at least 25 members have been reported to belong to S100 family in humans. Twenty-one of them are coded by genes clustered at chromosome locus 1q21, known as the epidermal differentiation complex (EDC) involving in epithelial-derived cell differentiation [31]. It has been reported some members of this family were overexpressed in various cancer types and their functions might involved metastasis and EMT [32]–[34]. The EDC contains two groups of consecutively distributed S100A genes. One group contains S100A1, S100A13, S100A14, S100A16, S100A2-S100A7, S100A15, S100A8, S100A12 and S100A9 and the other contains FLG, HRNR (S100A18), RPTN, TCHH, TCHH1 (S100A17), S100A11 and S100A10. The other genes belonging to the subfamilies of S100B, S100P, S100Z and S100G are, respectively, located at chromosome loci 21q22, 4p16, 5q14 and Xp22. It was been reported that S100A genes had a common origin in the molecular evolution while S100B, of S100P, S100Z and S100G had different origins, so S100A genes might have a universal conserved regulatory sequences [35]. Given their highly conserved Gli1 binding homologous sequences, it is speculated that they play similar role related to Gil1, though we cannot exclude the possibility that they have different functions unrelated to Gil1. Our results suggested that the expression of S100A genes family in PC cells has three manners: First, expressing and depending on Gli1; second, expressing but not depending on Gli1; third, not expressing. Combinating Gli1 enhancer prediction with microarray data, we speculated that Gli1 regulated S100A genes through the cis-acting elements might be a fundamental mode of regulation. Previous study had reported that S100A7 and S100A9 transcription were induced by GlI1 treatment in epidermal cells [25]. During evolutionary process some S100A genes expression might be turned off due to obtain unknown repressor sequence and then some of these genes might be turned on again but no longer were regulated by Gli1 as a result of obtaining additional cis-acting elements. This hypothesis may partly interpret why the expression of S100A family is characterized by tissue selectivity though it is still lack of sufficient evidence. Detailed comparative genomics studies between distinct species, as well as different S100A genes might help to farther understand the exact mechanisms. In our microassay and the ensuing real-time PCR studies, several S100A genes, including S100A2, S100A4, S100A6, S100A11, and S100A14, were upregulated in response to Gil1. Several of these S100A genes such as S100A2, A4 and A6 have been shown to be involved in pancreatic cancer. In this study the data of XChIP assays showed that transcription factor Gli1 bound to the regulatory sequences of S100A2, A4 and A6 genes respectively in AsPC-1 cells, which suggested that these genes might be cis-regulated by Gli1.

Studies have shown that the functions of S100 gene family members were complex and diverse and So far, only S100A4 was found to be closely associated with EMT process. Moreover, in previous studies we have proposed the hypothesis that S100A4 gene might be one of the key factors in EMT molecular network regulated by Shh-Gli1 signaling pathway in pancreatic cancer cells [19]. Therefore, S100A4 gene was used as an example to explore the relationship between Shh-Gli1 signals and S100 gene family. As a matter of fact, numerous studies have indicated that the S100A4 protein levels in cancer tissue, pancreatic juice and serum of patients with pancreatic cancer were significantly increased [36]–[38]. Some studies have implied that S100A4 could be used as a potential biomarker of pancreatic cancer [39]. However, why S100A4 gene was overexpressed selectively in PC was uncertain. Maybe S100A4 gene could be regulated by certain selectively activated signaling pathway at the same time in pancreatic cancer. In the present study, we for the first time found that the expression levels of S100A4 protein were correlated positively with activity of Shh-Gli1 signals in pancreatic cancer tissues and the Gli1 protein upregulated S100A4 mRNA through cis-activation manner in PC cells which established an exact molecular pathway from Shh-Gli1 signals to S100A4 in PC cells.

Although activation of Hh signals almost accompanied the occurrence of EMT, to date, there was no evidence that Gli1 directly regulated Snail/Slug transcription. It has been reported that S100A4 and E-cadherin were inversely regulated in several cell systems and S100A4 promoted the expression of the essential transcription factors, Twist and Snail, in the EMT process, as well as mesenchymal markers, including VIM and MMPs [40]–[42]. Our data demonstrated that the abnormal activated Hh signaling pathway promoted EMT at least partially through mediating S100A4 gene in PC cells. Since S100A4 was an important pro-metastatic factor in PC, the new connection of Shh with S100A4 might be one of the key links within the molecular network during EMT of PC cells.

In conclusion, these data suggested a model in which Shh-Gli1 signaling pathway promotes metastasis in pancreatic cancer by promoting target genes transcription. Although tumor metastasis are constantly exposed to various prometastic factors and there may be more than one target gene of Gli1 involving in metastasis in pancreatic cancer, our work provided what we believed to be a novel mechanistic insight on transcriptional regulation of metastatic responses induced by Shh-Gli1 signaling pathway in cancer. Our findings provide a new connection establishing between Shh-Gli1 signals and S100A4 gene, which could be a therapy target for pancreatic cancer.

## References

[1] Bosetti C, Bertuccio P, Negri E, La Vecchia C, Zeegers MP (2012) Pancreatic cancer: overview of descriptive epidemiology. Mol Carcinog 51(1): 3–13. 22162227 10.1002/mc.20785

[2] Vincent A, Herman J, Schulick R, Hruban RH, Goggins M (2011) Pancreatic cancer. Lancet 378(9791): 607–620. 21620466 10.1016/S0140-6736(10)62307-0 PMC3062508

[3] Loos M, Kleeff J, Friess H, Büchler MW (2008) Surgical treatment of pancreatic cancer. Ann N Y Acad Sci 1138: 169–180. 18837898 10.1196/annals.1414.024

[4] Lees C, Howie S, Sartor RB, Satsangi J (2005) The hedgehog signalling pathway in the gastrointestinal tract: implications for development, homeostasis, and disease. Gastroenterology 129(5): 1696–1710. 16285967 10.1053/j.gastro.2005.05.010

[5] Parkin CA, Ingham PW (2008) The adventures of Sonic Hedgehog in development and repair. I. Hedgehog signaling in gastrointestinal development and disease. Am J Physiol Gastrointest Liver Physiol 294(2): G363–367. 18063705 10.1152/ajpgi.00457.2007

[6] Quint K, Stintzing S, Alinger B, Hauser-Kronberger C, Dietze O (2009) The expression pattern of PDX-1, SHH, Patched and Gli-1 is associated with pathological and clinical features in human pancreatic cancer. Pancreatology 9(1–2): 116–126. 19077462 10.1159/000178882

[7] Onishi H, Kai M, Odate S, Iwasaki H, Morifuji Y (2011) Hypoxia activates the hedgehog signaling pathway in a ligand-independent manner by upregulation of Smo transcription in pancreatic cancer. Cancer Sci 102(6): 1144–1150. 21338440 10.1111/j.1349-7006.2011.01912.x

[8] Carpenter RL, Lo HW (2012) Hedgehog pathway and GLI1 isoforms in human cancer. Discov Med 13(69): 105–113. 22369969 PMC3632644

[9] Tang SN, Fu J, Nall D, Rodova M, Shankar S (2012) Inhibition of sonic hedgehog pathway and pluripotency maintaining factors regulate human pancreatic cancer stem cell characteristics. Int J Cancer 131(1): 30–40. 21796625 10.1002/ijc.26323 PMC3480310

[10] Singh BN, Fu J, Srivastava RK, Shankar S (2011) Hedgehog signaling antagonist GDC-0449 (Vismodegib) inhibits pancreatic cancer stem cell characteristics: molecular mechanisms. PLoS One 6(11): e27306. 22087285 10.1371/journal.pone.0027306 PMC3210776

[11] Li X, Ma Q, Xu Q, Liu H, Lei J (2012) SDF-1/CXCR4 signaling induces pancreatic cancer cell invasion and epithelial-mesenchymal transition in vitro through non-canonical activation of Hedgehog pathway. Cancer Lett 322(2): 169–176. 22450749 10.1016/j.canlet.2012.02.035 PMC3408048

[12] Bailey JM, Mohr AM, Hollingsworth MA (2009) Sonic hedgehog paracrine signaling regulates metastasis and lymphangiogenesis in pancreatic cancer. Oncogene 28(40): 3513–3525. 19633682 10.1038/onc.2009.220 PMC2910592

[13] Feldmann G, Fendrich V, McGovern K, Bedja D, Bisht S (2008) An orally bioavailable small-molecule inhibitor of Hedgehog signaling inhibits tumor initiation and metastasis in pancreatic cancer. Mol Cancer Ther 7(9): 2725–2735. 18790753 10.1158/1535-7163.MCT-08-0573 PMC2605523

[14] Danilov AV, Neupane D, Nagaraja AS, Feofanova EV, Humphries LA (2011) DeltaNp63alpha-mediated induction of epidermal growth factor receptor promotes pancreatic cancer cell growth and chemoresistance. PLoS One 6(10): e26815. 22053213 10.1371/journal.pone.0026815 PMC3203907

[15] Neupane D, Korc M (2008) 14-3-3sigma Modulates pancreatic cancer cell survival and invasiveness. Clin Cancer Res 14(23): 7614–7623. 19047086 10.1158/1078-0432.CCR-08-1366 PMC3142357

[16] Li Z, Sclabas GM, Peng B, Hess KR, Abbruzzese JL (2004) Overexpression of synuclein-gamma in pancreatic adenocarcinoma. Cancer 101(1): 58–65. 15221989 10.1002/cncr.20321

[17] Li D, Zhu J, Firozi PF, Abbruzzese JL, Evans DB (2003) Overexpression of oncogenic STK15/BTAK/Aurora A kinase in human pancreatic cancer. Clin Cancer Res 9(3): 991–997. 12631597

[18] Hejna M, Hamilton G, Brodowicz T, Haberl I, Fiebiger WC (2001) Serum levels of vasoactive intestinal peptide (VIP) in patients with adenocarcinomas of the gastrointestinal tract. Anticancer Res 21(2A): 1183–1187. 11396161

[19] Xu X, Zhou Y, Xie C, Wei SM, Gan H (2012) Genome-wide screening reveals an EMT molecular network mediated by Sonic hedgehog-Gli1 signaling in pancreatic cancer cells. PLoS One. 2012 7(8): e43119. 10.1371/journal.pone.0043119 PMC3416762 22900095

[20] Mencía N, Selga E, Rico I, de Almagro MC, Villalobos X (2010) Overexpression of S100A4 in human cancer cell lines resistant to methotrexate. BMC Cancer 10: 250. 20515499 10.1186/1471-2407-10-250 PMC2903526

[21] Xu XF, Guo CY, Liu J, Yang WJ, Xia YJ (2009) Gli1 maintains cell survival by up-regulating IGFBP6 and Bcl-2 through promoter regions in parallel manner in pancreatic cancer cells. J Carcinog 8: 13. 19736394 10.4103/1477-3163.55429 PMC2746911

[22] Bigelow RL, Chari NS, Unden AB, Spurgers KB, Lee S (2004) Transcriptional regulation of bcl-2 mediated by the sonic hedgehog signaling pathway through gli-1. J Biol Chem 279: 1197–1205. 14555646 10.1074/jbc.M310589200

[23] Loos M, Hedderich DM, Ottenhausen M, Giese NA, Laschinger M (2009) Expression of the costimulatory molecule B7-H3 is associated with prolonged survival in human pancreatic cancer. BMC Cancer 9: 463–473. 20035626 10.1186/1471-2407-9-463 PMC2808322

[24] Yu M, Gipp J, Yoon JW, Iannaccone P, Walterhouse D (2009) Sonic hedgehog-responsive genes in the fetal prostate. J Biol Chem 284(9): 5620–5629. 19095649 10.1074/jbc.M809172200 PMC2645820

[25] Kasper M, Schnidar H, Neill GW, Hanneder M, Klingler S (2006) Selective modulation of Hedgehog/GLI target gene expression by epidermal growth factor signaling in human keratinocytes. Mol Cell Biol 26: 6283–6298. 16880536 10.1128/MCB.02317-05 PMC1592816

[26] Seidel K, Ahn CP, Lyons D, Nee A, Ting K (2010) Hedgehog signaling regulates the generation of ameloblast progenitors in the continuously growing mouse incisor. Development 137(22): 3753–3761. 20978073 10.1242/dev.056358 PMC3049275

[27] Katoh Y, Katoh M (2009) Hedgehog target genes: mechanisms of carcinogenesis induced by aberrant hedgehog signaling activation. Curr Mol Med 9: 873–886. 19860666 10.2174/156652409789105570

[28] Katoh Y, Katoh M (2008) Hedgehog signaling, epithelial-to-mesenchymal transition and miRNA (review). Int J Mol Med 22(3): 271–275. 18698484

[29] Sipos B, Möser S, Kalthoff H, Török V, Löhr M (2003) A comprehensive characterization of pancreatic ductal carcinoma cell lines: towards the establishment of an in vitro research platform. Virchows Arch 442(5): 444–452. 12692724 10.1007/s00428-003-0784-4

[30] Matthaios D, Zarogoulidis P, Balgouranidou I, Chatzaki E, Kakolyris S (2011) Molecular pathogenesis of pancreatic cancer and clinical perspectives. Oncology 81(3–4): 259–272. 22116519 10.1159/000334449

[31] Henry J, Toulza E, Hsu CY, Pellerin L, Balica S (2012) Update on the epidermal differentiation complex. Front Biosci 17: 1517–1532. 10.2741/4001 22201818

[32] Nipp M, Elsner M, Balluff B, Meding S, Sarioglu H (2012) S100-A10, thioredoxin, and S100-A6 as biomarkers of papillary thyroid carcinoma with lymph node metastasis identified by MALDI imaging. J Mol Med (Berl) 90(2): 163–174. 21938494 10.1007/s00109-011-0815-6

[33] Mishra SK, Siddique HR, Saleem M (2012) S100A4 calcium-binding protein is key player in tumor progression and metastasis: preclinical and clinical evidence. Cancer Metastasis Rev 31(1–2): 163–172. 22109080 10.1007/s10555-011-9338-4

[34] Lukanidin E, Sleeman JP (2012) Building the niche: The role of the S100 proteins in metastatic growth. Semin Cancer Biol 22(3): 216–225. 22381352 10.1016/j.semcancer.2012.02.006

[35] Shang X, Cheng H, Zhou R (2008) Chromosomal mapping, differential origin and evolution of the S100 gene family. Genet Sel Evol 40(4): 449–464. 18558076 10.1186/1297-9686-40-4-449 PMC2674912

[36] Ai KX, Lu LY, Huang XY, Chen W, Zhang HZ (2008) Prognostic significance of S100A4 and vascular endothelial growth factor expression in pancreatic cancer. World J Gastroenterol 14(12): 1931–1935. 18350635 10.3748/wjg.14.1931 PMC2700408

[37] Ikenaga N, Ohuchida K, Mizumoto K, Yu J, Fujita H (2009) S100A4 mRNA is a diagnostic and prognostic marker in pancreatic carcinoma. J Gastrointest Surg 13(10): 1852–1858. 19653048 10.1007/s11605-009-0978-4

[38] Suemizu H, Monnai M, Ohnishi Y, Ito M, Tamaoki N (2007) Identification of a key molecular regulator of liver metastasis in human pancreatic carcinoma using a novel quantitative model of metastasis in NOD/SCID/gammacnull (NOG) mice. Int J Oncol 31(4): 741–751. 17786304

[39] Ansari D, Rosendahl A, Elebro J, Andersson R (2011) Systematic review of immunohistochemical biomarkers to identify prognostic subgroups of patients with pancreatic cancer. Br J Surg 98(8): 1041–1055. 21644238 10.1002/bjs.7574

[40] Zhang HY, Zheng XZ, Wang XH, Xuan XY, Wang F (2011) S100A4 mediated cell invasion and metastasis of esophageal squamous cell carcinoma via the regulation of MMP-2 and E-cadherin activity. Mol Biol Rep 2 39: 199–208. 10.1007/s11033-011-0726-1 21603862

[41] Boye K, Mælandsmo GM (2010) S100A4 and Metastasis A Small Actor Playing Many Roles. The American Journal of Pathology 176: 528–535. 20019188 10.2353/ajpath.2010.090526 PMC2808059

[42] Oida Y, Yamazaki H, Tobita K, Mukai M, Ohtani Y (2006) Increased S100A4 expression combined with decreased E-cadherin expression predicts a poor outcome of patients with pancreatic cancer. Oncol Rep 16(3): 457–463. 16865243

